# Supplementary material for: Blood-Based Markers for Skeletal and Cardiac Muscle Function in Eventing Horses before and after Cross-Country Rides and How They Are Influenced by Plasma Volume Shift
Source: Animals (Basel). 2023 Oct 5;13(19):3110. doi: 10.3390/ani13193110 (PMC10572052; doi:10.3390/ani13193110)
Supplement: Supplementary file 1 [file animals-13-03110-s001.zip › animals-2577452-supplementary.pdf]

animals

MDPI

Article

Blood-Based Markers for Skeletal and Cardiac Muscle Function in Eventing Horses before and after Cross-Country Rides and How They Are Influenced by Plasma Volume Shift

Johanna Giers [1,*], Alexander Bartel [2], Katharina Kirsch [3], Simon Franz Müller [4], Stephanie Horstmann [5] and Heidrun Gehlen [1]

Table S1. Blood-based biomarkers with reference ranges, units and median (25th/ 75th quantiles) per sampled time point.

Parameter	N	Missing	Reference range	Unit	Median (25th,75th)			
					Pre (n=55)	10 min (n=55)	30 min (n=55)	next morning (n=54)
Albumin	162	2	25 - 54	g/l	33.9 (32.5;35.5)	37.1 (35.4;39.1)	36.1 (34.6;38.1)	34.2 (33.0;35.6)
AST	162	2	< 250	U/l	169.8 (131.8;309.0)	n.m.	186.2 (144.5;338.8)	182.0 (141.3;323.6)
Calcium	162	2	2.5 - 3.4	mmol/l	3.0 (3.0;3.1)	n.m.	3.1 (3.1;3.2)	3.1 (3.0;3.1)
Chloride	162	2	95 - 105	mmol/l	100 (98;101)	n.m.	99 (96;100)	100 (99;101)
CK	162	2	< 190	U/l	115 (74;196)	n.m.	172 (106;269)	158 (95;222)
Creatinine	162	2	71 - 159	µmol/l	94 (85;104)	n.m.	125 (115;134)	103 (88;112)
cTnI	162	2	< 0.03	ng/ml	0.01 (0.01;0.02)	n.m.	0.02 (0.02;0.03)	0.04 (0.02;0.07)
HCT	164	0	30.0 - 40.0	%	36.8 (34.3;38.9)	54.4 (50.6;57.3)	47.3 (44.2;49.3)	36.7 (34.8;38.7)
Inorg. Phosphate	162	2	0.7 - 1.5	mmol/l	1.1 (0.9;1.3)	n.m.	0.8 (0.7;1.0)	1.1 (1.0;1.2)
Lactate	130	34	< 0.9	mmol/l	0.67 (0.58;0.76)	8.81 (5.45;15.14)	3.21 (1.93;5.42)	0.70 (0.64;0.88)
LDH	162	2	< 400	U/l	377.3 (270.7;413.0)	n.m.	436.0 (376.8;493.2)	417.1 (337.9;477.2)
Magnesium	162	2	0.5 - 0.9	mmol/l	0.8 (0.7;0.8)	n.m.	0.7 (0.7;0.8)	0.7 (0.7;0.8)
Potassium	162	2	2.8 - 4.5	µg/l	3.5 (3.0;3.8)	n.m.	3.5 (3.2;3.7)	3.6 (3.1;3.8)
RBC	164	0	6.40 - 10.40	$x10^{12}/L$	7.73 (7.37;8.12)	10.97 (10.40;11.67)	9.63 (9.12;10.27)	7.98 (7.73;8.25)
SDMA	162	2	< 0.75	µmol/l	0.39 (0.34;0.42)	0.43 (0.37;0.49)	0.42 (0.38;0.49)	0.38 (0.31;0.43)
Sodium	162	2	125 - 150	mmol/l	138 (137;139)	n.m.	139 (138;141)	139 (137;140)
TP	162	2	55 - 75	g/l	61.0 (59.3;64.0)	67.4 (63.9;69.3)	64.6 (62.7;66.4)	61.8 (59.1;64.3)
UREA	162	2	3.3 - 6.7	mmol/l	4.8 (4.2;5.5)	5.2 (4.7;5.8)	5.4 (4.7;5.9)	5.3 (4.6;5.8)

* Parameters in an alphabetical order; n.m. = not measured; The reference ranges given for the individual parameters were provided by the evaluating laboratory for the specific test used.

Table S2. Blood-based biomarkers with reference ranges, units and estimated mean from the mixed model (95 % confidence interval) per time point for unadjusted values.

Parameter	Reference range	Unit	EMM + 95 % confidence interval			
			Pre (n=55)	10 min (n=55)	30 min (n=55)	next morning (n=54)
Albumin	25 - 54	g/l	34.0 (32.9;35.2)	37.5 (36.3;38.6)	36.4 (35.3;37.6)	34.4 (33.3;35.6)
AST	< 250	U/l	229 (178;295)	n.m.	251 (195;316)	240 (186;309)
Calcium	2.5 - 3.4	mmol/l	3.0 (3.0;3.1)	n.m.	3.1 (3.1;3.2)	3.0 (3.0;3.1)
Chloride	95 - 105	mmol/l	100 (99;101)	n.m.	99 (97;100)	100 (100;102)
CK	< 190	U/l	132 (102;170)	n.m.	191 (148;245)	166 (129;219)
Creatinine	71 - 159	µmol/l	95 (88;102)	n.m.	124 (117;131)	99 (93;106)
cTnI	< 0.03	ng/ml	0.02 (0.01;0.04)	n.m.	0.03 (0.01;0.05)	0.07 (0.04;0.09)
HCT	30.0 - 40.0	%	36.4 (34.8;38.0)	54.3 (52.7;56.0)	46.6 (45.0;48.3)	36.5 (34.9;38.2)
Inorg. Phosphate	0.7 - 1.5	mmol/l	1.1 (1.0;1.2)	n.m.	0.8 (0.7;1.0)	1.1 (1.0;1.2)
Lactate	< 0.9	mmol/l	0.7 (0.5;0.9)	9.4 (7.4;12.0)	3.7 (2.9;4.8)	0.7 (0.6;0.9)
LDH	< 400	U/l	363 (321;405)	n.m.	437 (395;479)	418 (376;460)
Magnesium	0.5 - 0.9	mmol/l	0.7 (0.7;0.8)	n.m.	0.7 (0.7;0.7)	0.7 (0.7;0.8)
Potassium	2.8 - 4.5	µg/l	3.3 (3.1;3.6)	n.m.	3.5 (3.3;3.7)	3.4 (3.2;3.6)
RBC	6.40 - 10.40	$x10^{12}/L$	7.70 (7.41;7.98)	11.03 (10.74;11.31)	9.65 (9.36;9.94)	7.88 (7.59;8.17)
SDMA	< 0.75	µmol/l	0.377 (0.349;0.406)	0.422 (0.393;0.450)	0.430 (0.401;0.458)	0.370 (0.341;0.398)
Sodium	125 - 150	mmol/l	138 (137;139)	n.m.	139 (138;140)	138 (137;140)
TP	55 - 75	g/l	62.0 (60.6;63.5)	67.4 (65.9;68.9)	65.4 (63.9;66.9)	62.4 (60.9;63.9)
UREA	3.3 - 6.7	mmol/l	4.99 (4.53;5.46)	5.38 (4.91;5.84)	5.44 (4.97;5.91)	5.30 (4.83;5.77)

* Parameters in an alphabetical order; n.m. = not measured; The reference ranges given for the individual parameters were provided by the evaluating laboratory for the specific test used.
